# Validation of a transcutaneous bilirubin meter in Mongolian neonates: comparison with total serum bilirubin

**DOI:** 10.1186/1471-2431-13-151

**Published:** 2013-09-27

**Authors:** Moe Akahira-Azuma, Naohiro Yonemoto, Battsengel Ganzorig, Rintaro Mori, Shinichi Hosokawa, Takeji Matsushita, Bayasgalantai Bavuusuren, Enkhtur Shonkhuuz

**Affiliations:** 1 Department of Pediatrics, National Center for Global Health and Medicine, Tokyo, Japan; 2 Department of Epidemiology and Biostatistics, National Center of Neurology and Psychiatry, Translational Medical Center, Tokyo, Japan; 3 National Center for Maternal and Child Health of Mongolia, Ulaanbaatar, Mongolia; 4 Department of Health Policy, National Center for Child Health and Development, Tokyo, Japan

**Keywords:** Bilirubin, Diagnosis, Hyperbilirubinemia, Kernicterus, Neonate

## Abstract

**Background:**

Neonatal hyperbilirubinemia, especially kernicterus, can be prevented by screening for neonatal jaundice. The transcutaneous bilirubin (TcB) meter is a non-invasive medical device for screening neonates. The study aimed to investigate the validity of a TcB meter in a resource-limited setting such as Mongolia.

**Methods:**

Term and late preterm neonates from the National Center for Maternal and Child Health of Ulaanbaatar in Mongolia who met the inclusion criteria (gestational age ≥35 weeks, birth weight ≥2000 g, postnatal age ≤ 1 month) were enrolled in the study. We used a TcB meter, JM-103 to screen for neonatal jaundice. TcB measurements at the infant’s forehead and midsternum were performed within 3 h of obtaining samples for total serum bilirubin (TSB) measurement. We analyzed the correlation between TcB measurements and TSB measurements to validate the meter.

**Results:**

A total of 47 term and six late preterm neonates were included in the study. TcB measured by the meter at both the forehead and the midsternum showed a strong correlation with TSB measured in the laboratory. The correlation equations were TSB = 1.409+0.8655 × TcB (R^2^=0.78871) at the forehead, and TSB = 0.7555+0.8974 × TcB (R^2^=0.78488) at the midsternum. Bland-Altman plots and the Bradley-Blackwood test showed no significant differences between the two methods at all measured ranges of bilirubin. The mean areas under the curves of TcB at the forehead and midsternum at three TSB levels (>10 mg/dL, >13 mg/dL, >15 mg/dL) of TcB were greater than 0.9, and all had high sensitivity and specificity.

**Conclusions:**

This study established the validity of the JM-103 meter as a screening tool for neonatal jaundice in term and late preterm infants in Mongolia. Future studies are needed, including the establishment of a TcB hour-specific nomogram, for more effective clinical practice to prevent severe hyperbilirubinemia.

## Background

The guidelines of the American Academy of Pediatrics (AAP) [1] indicate that transcutaneous bilirubin (TcB) measurements are accurate in the diagnosis of neonatal jaundice, and can reduce the need for blood sampling. In addition, they recommend that all neonates undergo total serum bilirubin (TSB) or TcB measurements at least once before hospital discharge to assess their risk of hyperbilirubinemia.

Severe hyperbilirubinemia (kernicterus and, irreversible neurological sequelae) in newborns is preventable through appropriate follow-up, diagnosis, and treatment, such as phototherapy and exchange transfusions [2-5]. Neonatal jaundice is more prevalent in the Asian population [6], which may lead to a higher risk of developing kernicterus. A study in Hong Kong reported that 23.9% of Chinese newborns had a peak TSB>204 μmol/L (12 mg/dL) [7], whereas a study of Canada found that 6.7% of newborns (predominantly white) had a peak TSB ≥230 μmol/L (13.5 mg/dL) [8]. Setia et al. [9] reported that the risk of severe jaundice, requiring phototherapy, blood transfusion, or rehospitalization, was significantly elevated in infants of full East Asian parentage.

The second generation transcutaneous jaundice meter (JM-103, Konica Minolta, Osaka, Japan) is a non-invasive medical device that is easy to use at the bedside, delivers prompt measurements, and is minimally influenced by skin pigmentation. According to the manufacturer’s instructions, it can be used for infants up to 1 month of age. Yasuda et al. [10] found an excellent correlation (r=0.93) between TcB (measured by JM-103) and TSB among Japanese term neonates. This diagnostic test was further validated among several ethnic groups in North America [11,12], Europe [13], and Asia [14,15].

Neonatal hyperbilirubinemia, especially kernicterus, could be prevented in a resource-limited setting where phototherapy is available by screening for neonatal jaundice. In Mongolia, phototherapy is available at secondary care hospitals. If the doctors in the primary care clinics can identify neonatal jaundice and refer the neonates to the secondary care hospital promptly, the neonates can be treated with phototherapy. However, a TcB test as screening tool for neonatal jaundice has not yet been validated in Mongolia.

To investigate the validity of the TcB meter in a resource-limited setting, we compared bilirubin levels measured by the JM-103 meter with laboratory TSB levels in neonates in Mongolia.

## Methods

### Patients

We collected data on newborns (gestational age≥35 weeks, birth weight ≥2000 g, postnatal age ≤ 1 month) who were admitted to the Neonatal Pathological Unit in the National Center for Maternal and Child Health (NCMCH) of Ulaanbaatar in Mongolia between October 1, 2010 and January 31, 2011. NCMCH is one of the tertiary hospitals for children in Mongolia.

This study was approved by the NCMCH ethics committee in Mongolia and the National Center for Global Health and Medicine (NCGM) human investigation ethics committee in Japan. Oral informed consent was obtained from the patients’ parents or guardians and approved by each ethics committee.

### Measurements

Measurement of TSB levels was based on the clinical indications (e.g., jaundice, or poor feeding). Blood was obtained by venipuncture, and collecting tubes were shielded from exposure to light. TSB levels were measured by a photometric analyzer (DiaSys Diagnostic Systems, Holzheim, Germany) in the hospital clinical chemistry laboratories. TcB levels were obtained within 3 h of sampling for TSB measurement through the use of the transcutaneous jaundice meter, JM-103 (Konica Minolta, Osaka, Japan), by designated pediatricians. Of the three most commonly used TcB meters (JM-103, Bilicheck and BiliMed), JM-103 and Bilicheck (SpectRX, Norcross, GA, USA) have been found to be the most reliable screening tools for hyperbilirubinemia [16]. The principles of operating the JM-103 and its measurement techniques have been described previously [10]. Briefly, TcB was measured at both the forehead (uncovered) and the midsternum (covered) three times each, and the median value was designated as the TcB at each measurement site.

### Statistical analysis

Linear regression analysis was used to determine the association between the two methods of measurement. We used Bland-Altman plots [17] (mean and 95% confidence intervals [CI]) to determine whether the difference obtained between the two methods was stable at all measured ranges of bilirubin, and the equality of the mean values and variances was examined using the Bradley-Blackwood test [18]. The Bradley-Blackwood test is a measure of agreement and simultaneously tests the equivalence of means and variances of two paired measurements. Pearson correlation coefficients (*r*) and coefficient of determination (R^2^) were calculated for TSB and TcB at both sites. We also constructed receiver operating characteristics (ROC) curves [19] for JM-103 cutoff values (mg/dL) at TSB >10 mg/dL, >13 mg/dL, and >15 mg/dL. All data were analyzed using JMP statistical software version 9.0.2 (SAS Institute, Cary, NC, USA).

## Results

The study population consisted of 53 ethnic Mongolian neonates. The clinical characteristics (gestational age, birth weight, delivery mode, and postnatal day of measurement of TSB and TcB) of the study population are shown in Table 1. The postnatal day of measurement of TSB and TcB was distributed fairly evenly over a 1-month period postnatally.

**Table 1:** Clinical characteristics of the 53 ethnic Mongolian neonates

**Characteristics**		**n**	**(%)**
Birth Weight (g):	<2500 g	2	3.8
	2500-3499 g	29	54.7
	3500-4499 g	22	41.5
Gestational Age (week):	35-36 weeks	6	11.3
	37-40 weeks	43	81.2
	41-42 weeks	4	7.5
Delivery Mode:	Normal Vaginal	41	77.4
	Cesarean Section	12	22.6
Postnatal day of measurement of	Day 0-10	19	35.9
	Day 11-20	15	28.2
	Day 21-31	19	35.9

**Figure 1:**
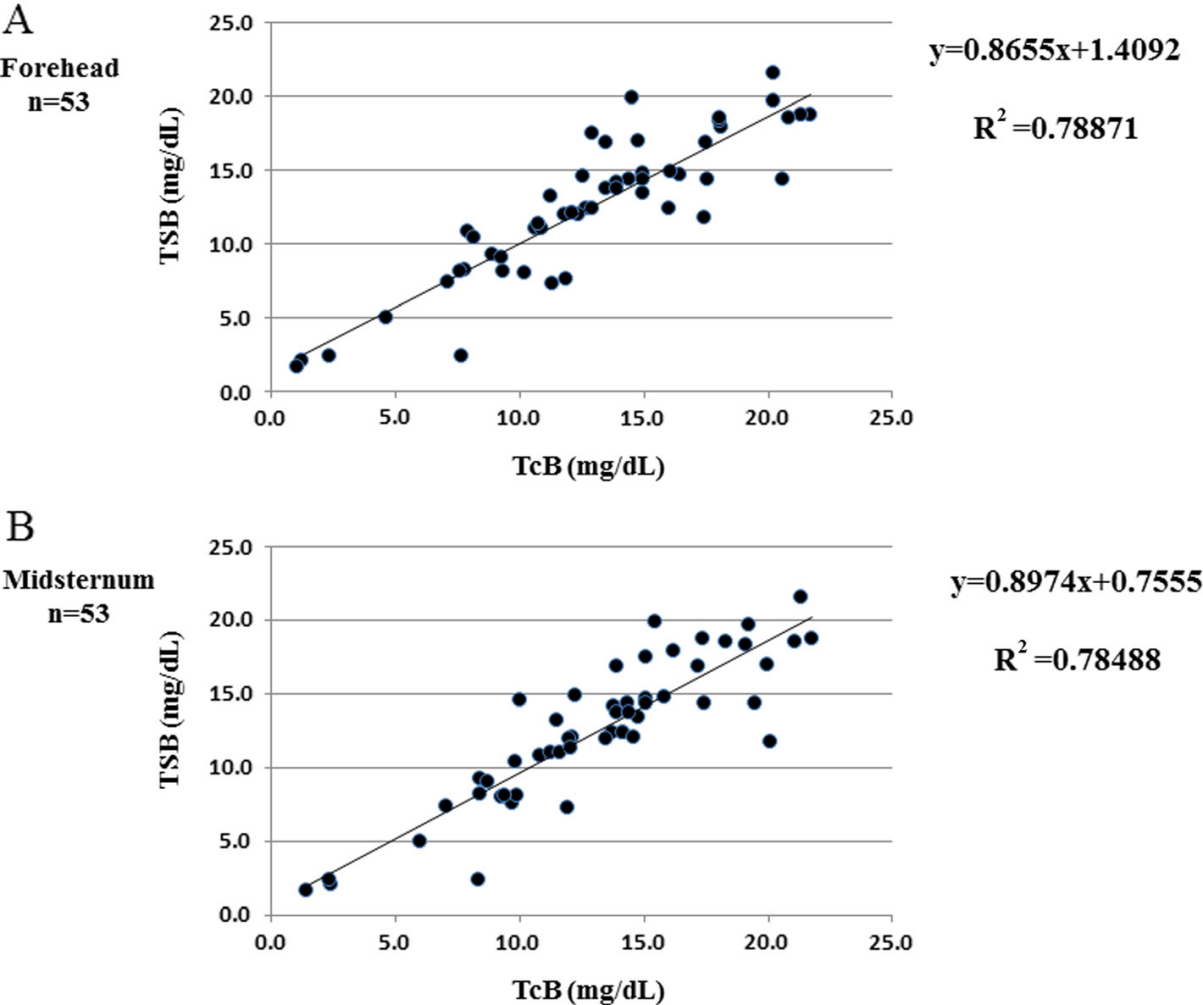
**Linear regression plots (solid lines) of JM-103 TcB *versus *TSB measurements at different measurement sites (A: forehead, B: midsternum) in the neonate.**

**Figure 2:**
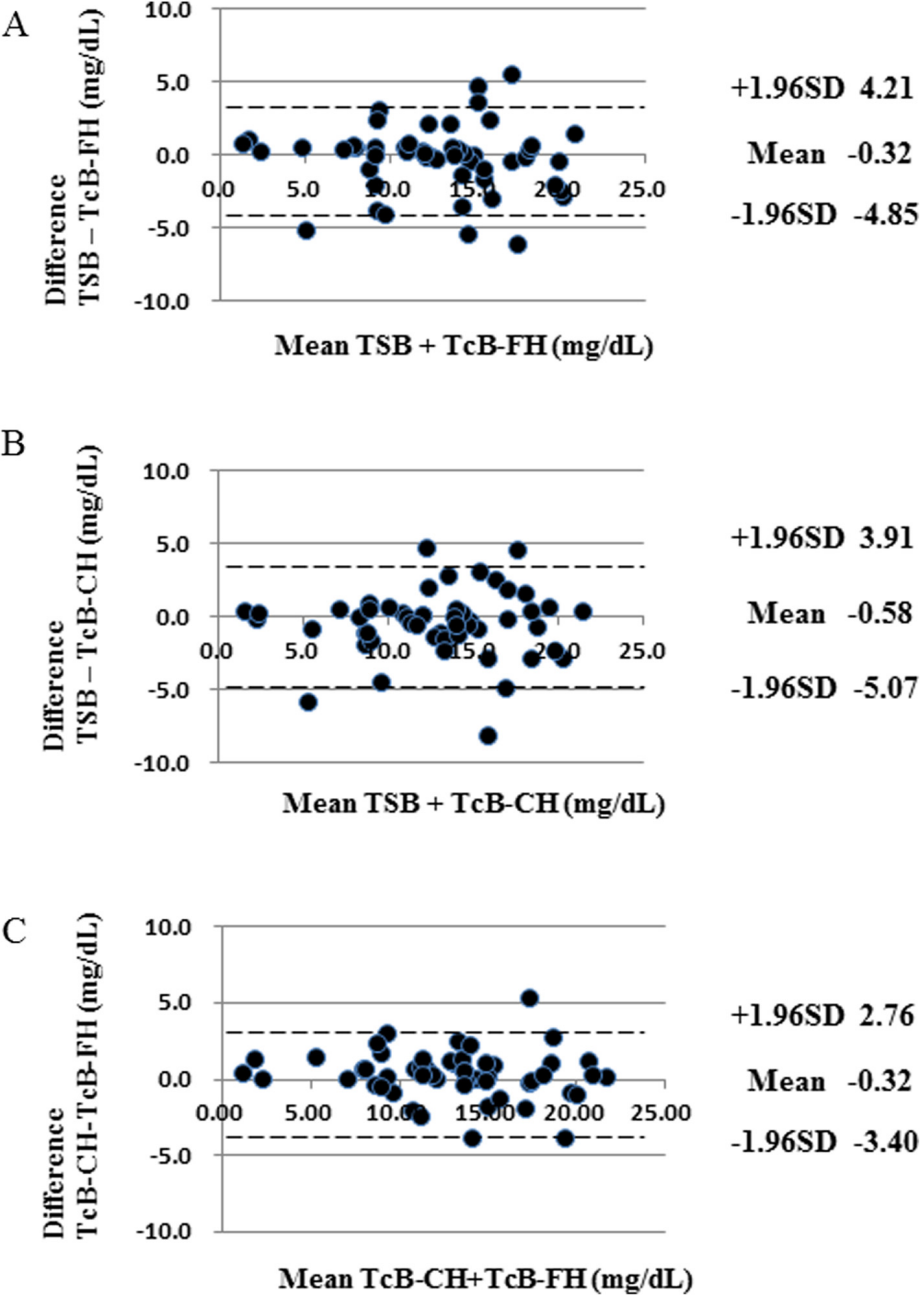
**Bland-Altman plots of TSB and TcB in the neonates.** The graphs show the difference in mean values between TSB and TcB (forehead) **(A)**, TSB and TcB (midsternum) **(B)**, and TcB (midsternum) and TcB (forehead) **(C)**. The P-value by the Bradley-Blackwood test was 0.315 in **(A)**, 0.073 in **(B)** and 0.247 in **(C)**. FH: forehead; CH: midsternum.

Table 2 summarizes mean (95% CI) levels of TSB and TcB (forehead and midsternum) and shows similarities between all three measurements. Linear regression analysis and Bland-Altman plots (mean and 95% CI) are shown in Figures 1 and 2. The Pearson correlation coefficients with respect to TSB were as follows: TcB forehead, 0.888 (R^2^=0.78871); and TcB midsternum 0.886 (R^2^=0.78488). The equality of the mean values and variances according to the Bradley-Blackwood test for all measured ranges of bilirubin are shown in Figure 2. The P-value for the comparison between TSB and TcB (forehead) was 0.315 (Figure 2A), for the comparison between TSB and TcB (midsternum) was 0.073 (Figure 2B), and for the comparison between TcB (forehead) and TcB (midsternum) was 0.247 (Figure 2C).

**Table 2:** Summary statistics of bilirubin measurements

**Variable**	**N**	**Mean**	**95% CI**	**SD**	**Minimum**	**Q1**	**Median**	**Q3**	**Maximum**
TSB	53	12.55	1.70-21.01	4.819	1.7	9.20	12.5	15.95	21.6
TcB Forehead	53	12.87	1.07-21.56	4.945	1.0	9.75	12.9	16.25	21.7
TcB Midsternum	53	13.12	1.72-21.63	4.755	1.4	9.85	13.7	16.00	21.8

TSB: total serum bilirubin; TcB: transcutaneous serum bilirubin; SD: standard deviation; CI: confidence interval.

**Figure 3:**
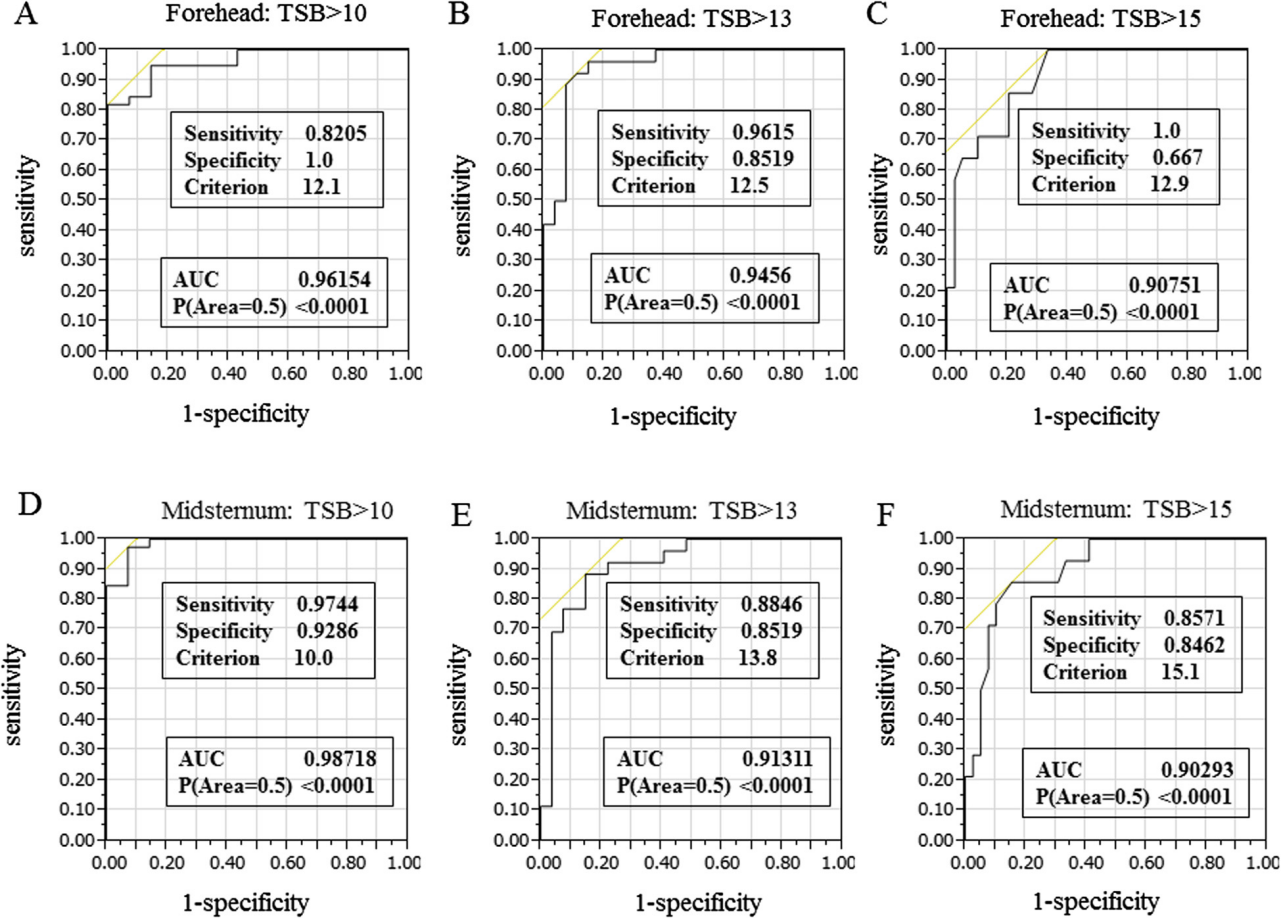
**Receiver operating characteristics curves for JM-103 cutoff values (mg/dL) at TSB >10 mg/dL, >13 mg/dL, or >15 mg/dL.** The areas under the curves were 0.962 **(A)**, 0.945 **(B)**, 0.908 **(C)**, 0.987 **(D)**, 0.913 **(E)**, and 0.903 **(F)**.

The ROC curves of mean TcB at TSB >10 mg/dL, TSB >13 mg/dL, and TSB >15 mg/dL are shown in Figure 3, and demonstrate graphically the acceptable specificity and sensitivity of mean TcB measurements at these threshold levels of TSB. All sites and levels showed the discriminatory power of the test. The areas under the curve (AUC) of forehead TcB levels were 0.961 at TSB >10 mg/dL, 0.945 at TSB >13 mg/dL, and 0.907 at TSB >15 mg/dL (Figure 3A, B, C). The AUC of midsternum TcB were 0.987 at TSB >10 mg/dL, 0.913 at TSB >10 mg/dL, and 0.902 at TSB >15 mg/dL (Figure 3D, E, F).

## Discussion

This study demonstrated a strong correlation between TcB and TSB measured at both the forehead and midsternum of ethnic Mongolian neonates. Since Yasuda et al. [10] published the accuracy of TcB measurement in Japanese neonates, several validations in diagnostic studies have been performed [11-16]. Regarding other Asian populations, a strong correlation between TcB and TSB was reported in a study in Thailand [14]. However, a study in Chinese newborns suggested that there was a significant difference between TcB and TSB [15]. In a previous study in an African American population, there was a lower correlation between the two measurements [11], but other populations, such as Caucasians and Europeans, showed a strong correlation [12,13,16]. The accuracy of TcB in our study was similar to that in some validated populations. A multi-ethnic population in the United States showed TcB AUC of 0.962 at 10 mg/dL TSB, 0.963 at 13 mg/dL, and 0.975 at 15 mg/dL [11], an Israeli population showed AUC of 0.958 at TSB 10 mg/dL, 0.971 at 13 mg/dL and 0.976 at 15 mg/dL, [13], and a Thai population showed AUC of 0.851 at TSB 10 mg/dL, 0.830 at 13 mg/dL, 0.958 at 15 mg/dL [14]. The study in the Israeli population reported a sensitivity and specificity of 89.4% and 100%, respectively, at TSB 10 mg/dL, of 93.6% and 90.5%, respectively, at 13 mg/dL, and 100% each at 15 mg/dL [13]. In the Thai population, sensitivity and specificity were 89.4% and 37.5%, respectively, at TSB 10 mg/dl, 93.6% and 65.7%, respectively, at 13 mg/dL, and 100% and 85.4%, respectively, at 15 mg/dL [14]. Our results validate the use of TcB as an alternative, quick and convenient measure of TSB in ethnic Mongolian neonates.

There are a number of previous reports of the comparison between TcB and TSB, with variability in study design. All hospital-based studies used convenience samples, but there were large variations in the number of infants (minimum 77 [10] and maximum 997 [15]). There were differences in inclusion criteria (e.g., healthy term or late preterm infants), in exclusion criteria (e.g., gestational age less than 35 weeks, receiving phototherapy, ABO incompatibility, Rh incompatibility, major congenital malformation, hemoglobinopathies or evidence of liver disease), in the TcB measurement site (e.g., forehead, midsternum, or both sites), in the number of measurements (1 to 5 times), and in ethnicity. Studies have been performed in Asian populations in Japan [10], Thailand [14], and China [15], and in mixed race countries such as the United States (white, African-American, East Asian, and Middle eastern) [11], Canada (Caucasian and Non-Caucasian) [12], Israel (Ashkenazi, Sephardic, and Ethiopian) [13], and Italy (Caucasian and West African) [16]. In addition, TSB was measured by different methods (e.g., photometric, colorimetric, direct spectrophotometry, and high performance liquid chromatography). Because of these variations, there were no comprehensive studies. Therefore, it was worthwhile to perform this validation study in a resource-limited setting like Mongolia. However, the limitations of our study were that i) the sample size was relatively small compared with previous studies; ii) the analyzed infants had medical indications for admission to a single hospital; and iii) the infants were relatively older than in previous reports. In addition, the Bradley-Blackwood test for all measured ranges of bilirubin did not clearly present equivalence, because our sample size was relatively small and had low statistical power.

As we had limited medical resources, we selected term and late preterm neonates who required re-admittance for visible jaundice and other medical reasons after discharge. We were allowed to take their blood samples, including TSB, for diagnosis and management. The 3-h time difference between sampling for TSB measurement and TcB measurement was relatively long compared with previous studies. However, this longer time period may not affect the differences between TSB and TcB. In most cases (n=45, 84.9%), we performed the measurements at postnatal 6 day or later (Table 1). TSB tends to increase on an hourly basis until day 4 or 5 after birth, then plateaus and decreases gradually [1].

Because the AAP recommends that TcB measurements are accurate and constitute a viable alternative to TSB that can reduce the need for blood sampling, a TcB hour-specific nomogram has been constructed in many countries [20-26]. DeLuca et al. [27] observed that TcB levels plateau and then decrease after 96 h of life in healthy neonates, with some differences across populations. Thus, it is necessary to create a Mongolian ethnicity-specific TcB nomogram in the near future.

The JM-103 meter had good validity. This tool is non-invasive and easy to use, gives prompt measurements, and is minimally influenced by skin pigmentation. Thus, it might afford the possibility of screening jaundice in a home visit setting. The TcB jaundice meter would be suitable for the early detection of subsequent hyperbilirubinemia. Therefore, we would need to create a TcB hour-specific nomogram to prevent severe hyperbilirubinemia in Mongolia.

## Conclusion

This study established the validity of the JM-103 meter as a screening tool for neonatal jaundice in term and late preterm Mongolian infants. However, future studies are needed to create a TcB hour-specific nomogram for more effective clinical practice to prevent severe hyperbilirubinemia.

## Abbreviations

AAP: American Academy of Pediatrics; AUC: Areas under the curve; CI: Confidence interval; CH: Midsternum; CV: Coefficient of variation; FH: Forehead; ROC: Receiver operating characteristics; SD: Standard deviation; TcB: Transcutaneous bilirubin; TSB: Total serum bilirubin.

## Competing interests

The authors declare that they have no competing interests.

## Authors’ contributions

MAA conceptualized and designed the study, carried out the initial analyses, drafted the initial manuscript, and approved the final manuscript as submitted. NY conceptualized and advised the analysis, reviewed the manuscript, and approved the final manuscript as submitted. BG designed the data collection instruments, coordinated and reviewed the manuscript, and approved the final manuscript as submitted. RM conceptualized and reviewed the manuscript, and approved the final manuscript as submitted. SH, TM reviewed the manuscript, and approved the final manuscript as submitted. BB designed the data collection instruments, coordinated and supervised data collection, reviewed the manuscript, and approved the final manuscript as submitted. SE reviewed the manuscript, and approved the final manuscript as submitted.

## Pre-publication history

The pre-publication history for this paper can be accessed here:

http://www.biomedcentral.com/1471-2431/13/151/prepub
